# A Comprehensive Review of *Andrographis paniculata* (Burm. f.) Nees and Its Constituents as Potential Lead Compounds for COVID-19 Drug Discovery

**DOI:** 10.3390/molecules27144479

**Published:** 2022-07-13

**Authors:** Aekkhaluck Intharuksa, Warunya Arunotayanun, Wipawadee Yooin, Panee Sirisa-ard

**Affiliations:** 1 Department of Pharmaceutical Sciences, Faculty of Pharmacy, Chiang Mai University, Chiang Mai 50200, Thailand; aekkhaluck.int@cmu.ac.th (A.I.); wipawadee.y@cmu.ac.th (W.Y.); panee.sirisa-ard@cmu.ac.th (P.S.-a.); 2 Kanchanabhishek Institute of Medical and Public Health Technology, Praboromarajchanok Institute, Nonthaburi 11150, Thailand

**Keywords:** *Andrographis paniculata*, lead compound, natural product, COVID-19, SARS-CoV-2, antiviral activity, anti-inflammatory activity, immunomodulatory activity

## Abstract

The COVID-19 pandemic has intensively disrupted global health, economics, and well-being. *Andrographis paniculata* (Burm. f.) Nees has been used as a complementary treatment for COVID-19 in several Asian countries. This review aimed to summarize the information available regarding *A. paniculata* and its constituents, to provide critical points relating to its pharmacological properties, safety, and efficacy, revealing its potential to serve as a source of lead compounds for COVID-19 drug discovery. *A. paniculata* and its active compounds possess favorable antiviral, anti-inflammatory, immunomodulatory, and antipyretic activities that could be beneficial for COVID-19 treatment. Interestingly, recent in silico and in vitro studies have revealed that the active ingredients in *A. paniculata* showed promising activities against 3CLpro and its virus-specific target protein, human hACE2 protein; they also inhibit infectious virion production. Moreover, existing publications regarding randomized controlled trials demonstrated that the use of *A. paniculata* alone or in combination was superior to the placebo in reducing the severity of upper respiratory tract infection (URTI) manifestations, especially as part of early treatment, without serious side effects. Taken together, its chemical and biological properties, especially its antiviral activities against SARS-CoV-2, clinical trials on URTI, and the safety of *A. paniculata*, as discussed in this review, support the argument that *A. paniculata* is a promising natural source for drug discovery regarding COVID-19 post-infectious treatment, rather than prophylaxis.

## 1. Introduction

Since 2019, Coronavirus disease (COVID-19), which is caused by severe acute respiratory syndrome coronavirus 2 (SARS-CoV-2), has emerged as a global challenge, particularly in terms of the rapid increase in critically ill patients, leading to morbidity and mortality. Up until May 2022, over 500 million infected cases and over 6 million deaths have been confirmed worldwide [1]. SARS-CoV-2 initiates its infection process via interaction with the human angiotensin-converting enzyme 2 (ACE2) receptors through its receptor-binding domain (RBD) [2], and uses the transmembrane protease serine 2 (TMPRSS2) for priming the spike protein [3]. COVID-19 patients can be asymptomatic or suffer from mild respiratory symptoms. Unfortunately, many patients suffering from SARS-CoV-2 infection subsequently developed acute respiratory distress syndrome (ARDS) and cytokine release syndrome (CRS), which led to life-threatening multi-organ failures that were considered to be the leading cause of death [4]. Although COVID-19 vaccinations have proved to be an effective prevention method that has helped to reduce the numbers of new cases and mitigate the severity of infected cases, the contagious nature of this virus and the emergence of new SARS-CoV-2 variants have given rise to concerns that this epidemic will be prolonged. Since no gold-standard therapeutics are available, researchers are urgently seeking possible solutions to tackle this pandemic situation. Several natural products and traditional medicines have been reviewed and considered promising prophylactic agents, treatments, or lead compounds for drug discovery due to their potential properties and tolerable toxicity [5]. In this review, we discuss *Andrographis paniculata* (Burm. f) Nees, a herbal medicine that has been used traditionally to alleviate flu and respiratory syndrome and has proven effective for its antiviral, anti-inflammatory, and immunomodulatory activity. Although the phytochemical and pharmacological profiles of *A. paniculata* have previously been reviewed [6,7,8], this article aims to provide valuable and up-to-date scientific data regarding *A. paniculata* and its constituents relating to COVID-19. The extract of *A. paniculata* and the andrographolide derivatives are among natural lead compounds that have been selected and evaluated in recent in silico and in vitro models for their potential in COVID-19 management.

**Figure 1:**
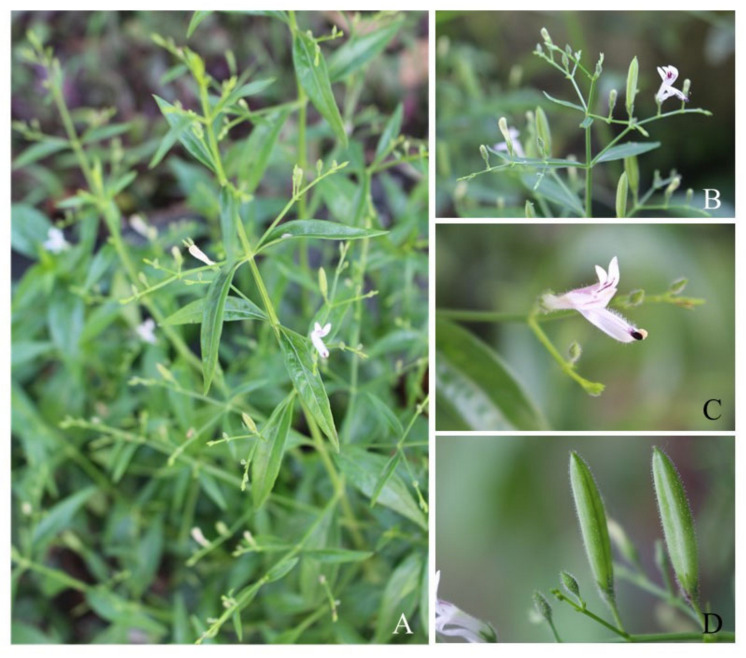
Morphological characteristics of *Andrographis paniculata.* (**A**): Aerial part, (**B**): fruits and flowers, (**C**): close-up of the flower, and (**D**): fruits.

*Andrographis paniculata* (Burm. f) Nees (synonyms *A. paniculata* var. *glandulosa* Trimen., *Justicia paniculata* Burm. f), belonging to the Acanthaceae family, is an annual herbaceous plant that is native to India and Sri Lanka; it has also been cultivated or naturalized in various other areas of the world (China, Cambodia, Indonesia, Laos, Malaysia, Myanmar, Thailand, Vietnam, and the Caribbean) as shown in Figure 1 [9]. It is commonly known as the “king of bitter”, or green Chiretta in English. It is also known as “Kalmegh” in Hindi, “Chanxinlian” in Chinese, “fah tha lai” in Thai, and “Hempedubumi” in Malaysian. The aerial part of *A. paniculata* has been effectively utilized as a medicinal herb in many traditional systems for centuries, used to cure fever and to treat the common cold, respiratory symptoms, diabetes, and skin infections [10]. *A. paniculata* is described in several official materia medica and national pharmacopeia, such as the Chinese herbal pharmacopeia, Indian herbal pharmacopeia, Thai herbal pharmacopeia and British herbal pharmacopeia, and appears in the United States pharmacopeia as a dietary supplement. Furthermore, the World Health Organization included *A. paniculata* as a medicinal plant in a WHO monograph on widely used medicinal plants that was intended to monitor quality control and herbal medicine usage [11]. In Thailand, the Ministry of Public Health has chosen *A. paniculata* as one of the essential medicinal plants included in the Thailand National List of Essential Medicines [12], used in hospitals and public health services.

## 2. Materials and Methods

This study was conducted by reviewing the published information in the national pharmacopeias, books, published articles, and electronic databases such as the Web of Science, Scopus, PubMed, Google Scholar, Elsevier, and so on. The extensive results were explored for subsequent analyses and summaries of the botanical and chemical profiles, as well as the scientific data, of *A. paniculata* and its related compounds in terms of its pharmacological properties in the context of COVID-19. Furthermore, we have also gathered information on the safety profiles of *A. paniculata* for this article.

## 3. Results

### 3.1. Traditional Uses

The aerial parts and roots of *A. paniculata* are utilized in many countries for different medicinal purposes, in Asia as well as Europe. The crude drug from *A. paniculata*, known as Kalmegh in India, has been commonly used as a primary component in more than 26 Ayurvedic formulations [10,13]. In addition, it is reported that the drug has been employed to treat all types of fever, particularly intermittent fevers [14]. Obtained from India and Southeast Asia for over century *A. paniculata* (Chuanxinlian in Chinese) has been used in Chinese medicine to clear heat and dampness; according to the traditional theory of Chinese medicine, it counters fire on account of its cooling properties [15]. In Southeast Asian countries, e.g., Thailand and Malaysia, it has been utilized for the treatment of diabetes and hypertension in Malaysian folk medicine [8]. Conversely, in Thai traditional medicine, *A. paniculata* has been given to patients suffering from fever and common cold symptoms. It is also used in Scandinavian countries for curing fevers and the symptoms of the common cold [10].

### 3.2. Chemical Constituents

**Figure 2:**
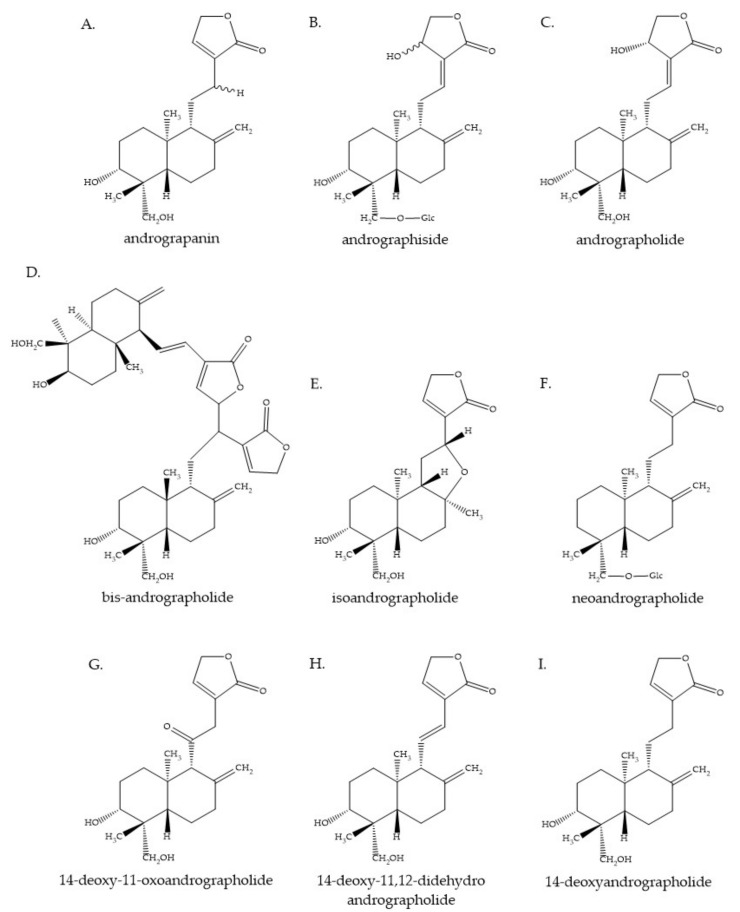
Some of the chemical constituents of *Andrographis paniculata.* (**A**): Andrograpanin, (**B**): andrographiside, (**C**): andrographolide, (**D**): bis-andrographolide, (**E**): isoandrographolide, (**F**): neoandrographolide, (**G**): 14-deoxy-11-oxoandrographolide, (**H**): 14-deoxy-11,12-didehydroandrographolide, and (**I**): 14-deoxyandrographolide.

The chemical substances that are found in *A. paniculata* have been studied extensively, as shown in Table 1. It is reported that the plant contains many diterpenoids, lactones, and flavonoids. Nevertheless, the concentration and composition of its phytochemicals vary, according to geography, plant parts, season, and phenological growth stage [16]. The main active compound of *A. paniculata* is andrographolide, which is found in the whole plant, leaves, stem, and roots. The characteristics of andrographolide are a colorless and bitter *ent*-labdane diterpene lactone substance [17]. Actually, this substance was first isolated by Boorsma, who named it andrographide. Then, in 1911, Gorter proved the structure of andrographolide and gave it that name (Figure 2C) [17,18]. Andrographolide can be extracted from all parts of the plant but it is most highly concentrated in the leaves [10,19,20,21,22,23]. There are several studies proving that the highest amount of andrographolide is found in the vegetative stage before flowering (about 130 days after initial cultivation) [22,24,25]. Andrographolide possesses various pharmacological properties, such as anti-allergic, anti-bacterial, anticancer, anti-diabetic, anti-dyslipidemic, anti-inflammatory, anti-leishmanial, anti-viral, antipyretic, analgesic, hepatoprotective, and neuroprotective activities [26,27,28,29,30,31,32,33]. The other *ent*-labdane diterpenoids from this plant, i.e., neoandrographolide (Figure 2F), isoandrographolide (Figure 2E), 14-deoxy-11, and 12-didehydroandrographolide (Figure 2H), also exhibit pharmacological activities [24]. In addition, the findings of this study indicate that a number of flavonoids have been reported in the past three decades. The isolation and elucidation of flavonoids were mostly performed using the root, but they were also found in the aerial parts of the plant. Previous studies revealed that the flavonoids show biological activity, including anti-proliferative and anti-platelet aggregation properties [24,34,35]. Besides this, xanthone, quinic acid and its derivative, sitosterol, and polysaccharide were reported as minor chemical substances found in *A. paniculata* [36,37,38].

### 3.3. Pharmacological Activities: Anti-SARS-CoV-2 and Other Anti-Virus

Since severe acute respiratory syndrome coronavirus 2 (SARS-CoV-2) has been identified as the causative pathogen of the COVID-19 outbreak, antiviral drugs have been at the top of the list for COVID-19 management, to reduce viral shedding and control the spread. To date, there is no specific antivirus against SARS-CoV-2. Certain conventional antiviral agents, i.e., lopinavir/ritonavir, favipiravir, and remdesivir have been utilized in many countries during the pandemic [84]. However, their effectiveness, specificity, limitations, and the adverse effects of existing treatments that have occurred in several cases have led to an urgent need for effective drugs for the prevention and treatment of COVID-19. Many studies had previously confirmed the virucidal activity of *A. paniculata* against different viral strains, including the influenza A virus (IAV), human immunodeficiency virus (HIV), Hepatitis B and C, Herpes Simplex virus I, Epstein-Barr virus, human papillomavirus, and Chikungunya virus [85].

#### 3.3.1. In Silico Analysis of Potential Anti-SARS-CoV-2 Agents from *A. paniculata* Phytochemicals

Due to the rapid spread of COVID-19, scientists around the world are racing against time to find potential anti-SARS-CoV-2 agents. The in silico approach is often used to identify drugs or potential compounds, to save time and costs. Several studies have employed in silico approaches, particularly molecular docking, and molecular dynamics (MD) simulations, to study the effects of AP phytochemicals on SAR-CoV-2’s vital proteins and the proteins involved in the process of infecting the human body. In this paper, we will focus on the in silico analysis of *A. paniculata* phytochemicals on five potential drug targets: the spike (S) glycoprotein, 3-chymotrypsin-like protease (3CLpro), papain-like protease (PLpro), and RNA-dependent RNA polymerase (RdRp) of SARS-CoV-2 and human angiotensin-converting enzyme 2 (hACE2) [86].

The SAR-CoV-2 S-glycoprotein, a trimeric class I fusion protein, is marked because it plays a pivotal role in SARS-Cov-2 attachment via its binding to hACE2. S-glycoprotein contains two domains, S1 and S2. The S1 receptor-binding domain (RBD) binds to the peptidase extracellular domain (PD) of hACE2; then, cleavage by the host proteases at the S1–S2 inter-domain protease site leads to the fusion of the S2-mediated virus and host cell membrane [87,88]. The S-glycoprotein is a key target for vaccines, therapeutic antibodies, small-molecule drugs, and diagnostics [86]. Recently, Hiremath et al. conducted an in silico docking analysis using AutoDock Vina software on 14 *A. paniculata* phytochemicals, including andrographolide, in terms of their being SAR-CoV-2 inhibitors and revealed that various *A. paniculata* phytochemicals can bind SARS-CoV-2 proteins, especially the RBD of the S-glycoprotein. Isoandrographolide (Figure 2E) is the best substance for binding to the S-glycoprotein, with the lowest binding energy (−9.1 kcal/mol). Besides this, other *A. paniculata* phytochemicals, comprising 19-O-acetyl-14-deoxy-11,12-didehydroandrographolide, neoandrographolide (Figure 2F), and 5-hydroxy-7,8,2′,5′-tetramethoxy flavone, also showed low binding energy to the S-glycoprotein. The results indicated that andrographolide (Figure 2C), the major active ingredient, binds to S-glycoprotein with a binding energy of −7.9 kcal/mol. The findings of Hiremath et al. also showed that there are no significant differences in the binding affinities of these substances with closed- and open-state S-glycoprotein [89]. Previously, Lakshmi et al. conducted their study in a similar way, but instead added a 200 ns MD simulation and used the same open-state S-glycoprotein structure (PDB ID: 6VYB). This work only studied three of the major active ingredients of *A. paniculata*: andrographolide, bis-andrographolide, and caffeic acid. The results showed that andrographolide is one of the top 10 substances that bind to open-state S-glycoprotein, out of 47 bioactive compounds taken from ten traditional medicinal plants [82]. The research by Dey et al. on the effect of andrographolide and 14-deoxy-11,12-didehydroandrographolide (Figure 2H), using AutoDock 4.0 software, showed that both substances bind equally well to S-glycoprotein and tend to be more binding to S-glycoprotein than 3CLpro and PLpro [90]. From the above results, it can be seen that the best bioactive compound under consideration that is likely to inhibit the S-glycoprotein is isoandrographolide. However, andrographolide, a major active ingredient, also tends to bind and inhibit the activity of S-glycoprotein.

The next protein target that should be mentioned after S-glycoprotein is the metallopeptidase enzyme, hACE2. As previously mentioned, the research shows that the inhibition of hACE2 or the inhibition of the binding of the S-protein to the PD of hACE2 is a promising method of treating SAR-CoV-2 infection. The study by Alazmi et al. explores the same direction as another study by Srivastava et al. Both studies use molecular docking and continue with MD simulation, but they each used a different 3D structure of hACE2 (PDB ID: 6M17) and the model, based on the BLAST parameters for the Alazmi and Srivastava studies, respectively. However, both studies showed that andrographolide is a promising hACE2 inhibitor. Alazmi et al. found that andrographolide is the best compound, out of over 200,000 natural compounds, for hACE2 interaction, demonstrating the lowest binding energy, as calculated using the MM-PBSA/MM-GBSA tool. Likewise, Srivastava and colleagues reported that andrographolide is one of the best three compounds out of the 30 natural and synthetic compounds that demonstrate in vitro and in vivo antiviral activity [81,91].

The development of SAR-CoV-2 protease inhibitors is another potential method for treating SAR-CoV-2 infection, in particular, two cysteine proteases, the 3-chymotrypsin-like protease (3CLpro) or main protease (Mpro) and the papain-like protease (PLpro). These proteases take part in the cleavage of polyproteins to produce the non-structural proteins 2–16 (nsp2−16), which are involved in the replication-transcription complex and are critical for viral replication and transcription in the host cell [86]. Since the structures of 3CLpro and Plpro have been identified and are in the Protein Data Bank (PDB), many studies have been used on these structures to study the binding between the AP phytochemicals and the active site of these proteases. Sukardiman et al. found that among 45 phytochemicals from *A. paniculata*, the flavonoid glycosides 5,4′-dihydroxy-7-*O*-β-d-pyran-glycuronate butyl ester and andrographolide glycoside 3-*O*-β-d-glucopyranosyl-andrographolide showed good binding energy with 3CLpro and the highest similarity of interaction types with amino acid residues, compared to the co-crystal ligands of structure PDB ID: 6LU7 and indinavir, an HIV protease inhibitor [92]. Other substances, including andrographolide, bis-andrographolide (Figure 2D), andrographiside (Figure 2B), andrograpanin (Figure 2A), and neoandrographolide (Figure 2F) have also been reported to bind to 3CLpro [82,93,94,95,96,97]. Interestingly, the docking analysis conducted by Shi et al. showed that andrographolide binds to 3CLpro with a covalent linkage, which is an ideal springboard for developing cysteine protease inhibitors [97]. Similarly, a number of *A. paniculata* phytochemicals have been studied for their properties as PLpro inhibitors. However, previous studies have not been used on the SAR-CoV-2 PLpro structure, but instead used the PLpro structures of other coronaviruses or PLpro structures developed from homology modeling. Therefore, the accuracy of the study results is one of the important factors that should be considered, since using a PLpro protein structure from another coronavirus yields less accurate results than using the PLpro protein structure of SAR-CoV-2 itself. Elsewhere, 14-deoxy-11,12-didehydroandrographolide has been reported to be better than andrographolide for binding to PLpro, as analyzed by molecular docking [90]. Moreover, Murugan et al. also reported that neoandrographolide not only binds to 3CLpro but also binds to PLpro better than lopinavir, an HIV protease inhibitor [95]. Nevertheless, further in silico studies of the effects of *A. paniculata* phytochemicals using specific SAR-CoV-2 PLpro structures are needed.

The last protein drug target of SAR-CoV-2 infection discussed in this article is RdRp. RdRp or nsp12 is a crucial polymerase enzyme for genome replication and the gene transcription of SAR-CoV-2. as well as in other RNA viruses. This enzyme is the drug target of remdesivir, a nucleotide prodrug of an adenosine analog and a drug that is currently being used to treat patients with SAR-CoV-2 infection [86,98,99]. The molecular dockings and molecular dynamics simulation analysis of Sharma et al. showed that 5 bioactive compounds from *A. paniculata*, namely, andrographolide, hydroandrographolide, isoandrographolide, neoandrographolide, and oxoandrographolide, bind to RdRp but are not as successful as 3CLpro. The study by Sharma has the advantage of using the specific RdRp structure of SAR-CoV-2 (PDB ID: 6M71), while other structures were not used. Therefore, Sharma’s study offers a more realistic reflection of RdRp behavior [100]. Another study by Murugan et al., who used the homology model of RdRp, showed contradictory results. Neoandrographolide was reported as a potential compound for treating SAR-CoV-2 infection and binds to RdRp better than remdisevir and the main protease [95].

#### 3.3.2. In Vitro Studies of the Anti-SARS-CoV-2 Activity of *A. paniculata* Extract and Its Components

Although there are many in silico studies of individual small molecules from *A. paniculata* regarding anti-SARS-CoV-2 activity, there are limitations in vitro and a lack of in vivo studies. Sa-ngiamsuntorn and colleagues from Thailand recently published the results of studies on the anti-SARS-CoV-2 activity of the ethanolic extracts of *A. paniculata* and andrographolide in human lung epithelial cells. The results showed that *A. paniculata* ethanolic extract and andrographolide could inhibit the infectious virion production, with IC_50_ of 0.036 µg/mL and 0.034 µM, respectively [101], while a previous study by Shi et al. that studied its activity, both in silico and in vitro, found that andrographolide could inhibit the 3CLpro activities of SARS-CoV-2, with an IC_50_ of 15 µM [97]. Both studies show that andrographolide is more successful in inhibiting 3CLpro than in inhibiting infectious virion production. It can be surmised that andrographolide has multiple mechanisms of action and is also involved in multiple steps of the viral life cycle, as predicted by the in silico studies.

The review above indicates that the ethanolic extracts of *A. paniculata* and andrographolide are effective in inhibiting the production of the infectious virion and could be synergistic with modern drugs. In terms of the use of *A. paniculata* extracts and andrographolide as a prophylactic agent to prevent viral entry into the host cells, although in silico studies have demonstrated that several compounds extracted from *A. paniculata* could bind to human ACE2, further in vitro or in vivo studies should confirm this.

#### 3.3.3. Antiviral Activity

The traditional use of *A. paniculata* for respiratory infections had led to both in vitro and in vivo studies on anti-influenza activity. An in vitro assay demonstrated that andrographolide and its derivatives were active against various subtypes of Influenza A (IAV) viruses, including H1N1, H3N2, H9N2, and H5N1 [102,103,104,105]. Andrographolide was reported to ameliorate the H1N1 virus-induced cell mortality of an infected human bronchial epithelial cell line (16HBE) by inhibiting the viral-induced activation of the retinoic acid-inducible gene-I (RIG-I)-like receptor (RLR) signaling pathway [102]. Cai et al. found that 14-deoxy-11,12-dehydroandrographolide (DAP), another major constituent of *A. paniculata*, was more active than andrographolide against IAV H5N1 in vitro by means of inhibiting viral replication, restraining the nuclear export of vRNP complexes, and attenuating the immune dysregulation induced by virus infection [105]. An in vivo study conducted using the prophylaxis and treatment model revealed that AL-1 (14-a-lipoyl andrographolide), a synthetic derivative of andrographolide, was a potent antiviral agent against IAV subtypes H1N1, H9N2, and H5N1 by interfering with viral hemagglutinin and blocking viral binding to cellular targets [103].

Inhibitory activity against the HIV virus in the H9 cell line of *A. paniculata* aqueous extract was reported at nontoxic concentrations, with the growth of the H9 cells [106]. Andrographolide, a diterpene lactone compound, served as the main active component of its anti-HIV effect, along with its derivatives, such as 14-deoxy-11,12-didehydroandrographolide, dehydroandrographolide succinic acid monoester (DASM), the oxime of 3-keto derivative, amide derivatives of andrographolic acid, and the 12-ester of 12-hydroxy-14-deoxy-13,14-dehydroandrographolide [48,107,108]. One proposed mechanism involved the proprotein convertases -1 and -7 and the extract’s furin inhibition capacity, leading to the suppression of the proteolytic processing of glycoprotein gp160 by HIV, which is PC-mediated [109]. Moreover, the semisynthetic derivatives of andrographolide were evaluated in vitro, revealing that the 3-nitrobenzylidene derivative was more active against HIV, while the 2′,6′-dichloro-nicotinoyl ester derivative showed a higher therapeutic index compared to andrographolide. Computational studies exhibited inhibition capacity regarding andrographolide and its semisynthetic analogs on the gp120-mediated cell fusion of HL2/3 cells (expressing gp120 on its surface) with TZM-bl cells (expressing CD4 and co-receptors CCR5 and CXCR4) [107]. A phase I clinical trial using 10 mg/kg andrographolide was conducted in HIV patients; it showed no significant decrease in viral load but a significant increase in the CD4+ lymphocyte level, which may be a result of the inhibition of HIV-induced cell-cycle dysregulation [110].

The anti-herpes simplex virus 1 (HSV-1) activity of andrographolide and its derivatives, including neoandrographolide, 14-deoxyandrographolide (DAD), 14-deoxy-11,12-didehydroandrographolide, and 3,19-isopropylideneandrographolide (IPAD), was revealed in the in vitro studies, which suggest that these compounds possessed inhibitory effects on viral entry and replication [111,112]. The ethanolic extract of *A. paniculata* and the major compound andrographolide was reported to be active against the Epstein–Barr virus (EBV) by inhibiting the expression of EBV lytic proteins, transcription factors (Rta and Zta), and early antigen diffuse (EA-D), leading to the prevention of mature viral particles produced during the viral lytic cycle in P3HR1 cells [113]. Andrographolide and its dehydroandrographolide derivatives exhibited anti-HBV activity by inhibiting the secretion of the viral envelope antigen, HBsAg and HBeAg, as well as HBV DNA replication [77]. The anti-hepatitis C virus (HCV) of andrographolide was evaluated, showing that andrographolide could suppress HCV replication via the nuclear factor erythroid 2-related factor 2 (Nrf2)–mediated HO-1 (Nrf2–HO-1) signaling pathway, by activating p38 MAPK phosphorylation. Moreover, it acted as an adjuvant to the standard therapy IFN-α, an inhibitor targeting the HCV NS3/4A protease (telaprevir), and NS5B polymerase (PSI-7977) [114]. An in silico study demonstrated that andrographolide strongly and stably interacted with HCV NS3-4A protease and two drug-resistant mutants, namely, R155K and D168A, which had the least energy compared to a conventional medicine, Asunaprevir [115]. Andrographolide and 14-deoxy-11-oxoandrographolide (Figure 2G) possessed significant inhibitory activity against dengue viral protein NS5 in silico [116]. An in vivo assay confirmed the dengue viral inhibitory property of the methanolic and ethanolic extracts of *A. paniculata* [117,118]. The anti-Chikungunya virus (CHIKV) activity of andrographolide was examined in vitro, demonstrating an inhibitory effect on CHIKV infection by inhibiting protein synthesis and viral genome replication [119]. Gupta et al. confirmed the antiviral activity of andrographolide against CHIKV in vitro and in vivo, proposing that the inhibition of virus propagation and virus-induced inflammation, as well as the activation of the host’s innate immunity, might serve as mechanisms of action for anti-CHIKV [120]. Moreover, andrographolide and its derivatives, namely, 14-deoxy-11,12-didehydroandrographolide (14-DDA) and 3,19-isopropylidene andrographolide (IPAD), were active against human papillomavirus-16 (HPV16) infection, which is a high-risk human papillomavirus (HR-HPV) subtype causing cervical cancer; this is due to their abilities to inhibit E6 oncoprotein expression and restore p53 expression [121,122]. The overall virucidal bioactivity of *A. paniculata* against a diverse group of viruses from various families via different mechanisms has suggested that this plant has the potential for novel antiviral drug discovery.

### 3.4. Pharmacological Activity Relating to the COVID-19 Illness

#### 3.4.1. Immunomodulatory Activity

SARS-CoV-2 infection can cause many different clinical features with varying degrees of severity, ranging from mild to life-threatening, depending on the individual’s immune system. Innate and adaptive immune responses are known to play an important role in repelling viral invaders. In addition, an imbalanced or impaired immune system may contribute to the development of disease manifestations, in terms of severity or opportunistic infection [123]. Hence, an immunity-boosting agent could be a promising strategy to combat or promote the effective treatment of SARS-CoV-2 infection.

*A. paniculata*, along with its constituents, diterpene lactones, have been reported to exert immunomodulatory activities via numerous pathways for over two decades. It has been proposed that the immunostimulant properties of *A. paniculata* are responsible for a wide range of pharmacological effects, especially its anti-infectious, anti-inflammatory, and anticancer activity [10,48]. In 1993, Puri et al. revealed the in vivo immunostimulant activity of *A. paniculata* ethanolic extract and the purified diterpenes, andrographolide, and neoandrographolide. The stimulation of both antigen-specific and nonspecific immune responses was observed, resulting in the enhancement of humoral and cell-mediated immune responses to sheep red blood cells (SRBC), as well as the macrophage migration index (MMI) phagocytosis and the proliferation of splenic lymphocytes in treated mice. It was noteworthy that the ethanolic extract was more active than purified andrographolide, suggesting that other constituents in *A. paniculata* might lead to a synergistic effect toward the stimulant activity of ethanolic extract [124]. Correspondingly, Wang et al. found that andrographolide exhibited an in vitro and in vivo modulatory effect on both innate and adaptive immune responses by regulating macrophage phenotypic polarization and Ag-specific antibody production, as well as antigen-specific IL-4-producing splenocytes. In this study, andrographolide showed an inhibitory effect on the phosphorylation of ERK 1/2 (the MAPK signaling pathway) and AKT (the PI3K signaling pathway) in macrophages treated with andrographolide, suggesting that the ERK 1/2 and AKT pathways may contribute to the regulatory activity of andrographolide on macrophage activation and polarization [125].

In addition, the immune-boosting activity of three diterpene compounds isolated from a methanolic extract, i.e., andrographolide (Figure 2C), 14-deoxyandrographolide (Figure 2I), and 14-deoxy-11,12-didehydroandrographolide (Figure 2H), at a low concentration were reported in an in vitro study showing an enhancement of the proliferation of human peripheral blood lymphocytes (HPBLs) and interleukin-2 (IL-2) induction in HPBLs [126]. This study supports the finding in a study of normal and type-2 diabetic mice, which revealed that the methanolic leaf extract of *A. paniculata* led to the increment of blood and splenic lymphocyte count, as well as peritoneal macrophage count [127]. These results are consistent with an in vitro study showing that the ethanolic extract of *A. paniculata,* which contains andrographolide as a major component, was able to induce lymphocyte cell proliferation at low concentrations (1–16 μg/mL) [128]. A study in male albino rats also showed that the chronic administration of *A. paniculata* leaf aqueous extract, at 250 mg/kg and 500 mg/kg, significantly increased white blood cell, lymphocyte, and monocyte counts, as well as serum IL-6 and TNF-α, and decreased the neutrophil and eosinophil counts. However, in this study, there was evidence that a 1 g/kg dosage of *A. paniculata* aqueous extract might contribute to anemia, multiple myeloma, and autoimmunity [129].

The immunomodulatory effects of mixtures and commercial medications containing *A. paniculata* and diterpene active compounds were investigated in several studies. In China, an in vitro study on LianBiZhi (LBZ), an injection including andrographolide as a primary active compound, confirmed that andrographolide had effects on both antigen-specific and nonspecific immune function by enhancing IFN-alpha, IFN-gamma, TNF-alpha, and IL-8 in peripheral blood mononuclear cells (PBMCs), as well as macrophage phagocytotic function and augmented natural killer cell cytotoxicity, damaging the K562 cell lines [130]. In Europe, the commercial preparation containing *A. paniculata* extract called “Kan Jang” and pure andrographolide was reported to inhibit the spontaneous proliferation of human peripheral blood lymphocyte (PBL) and stimulated the formation of interferon-gamma (INF-γ), tumor necrosis factor-alpha (TNF-α), and certain immune activation markers, such as neopterin (Neo) and β-2-microglobulin (β2MG), which could support the anti-infectious and anti-inflammatory effects of these agents. When at the equivalent amount of andrographolide, Kan Jang showed better immunostimulant activity than pure andrographolide, which could also be explained by the synergistic effect of the active components in *A. paniculata* [131]. Moreover, HN-02, a mixture containing andrographolides andrographolide (88 ± 5%) plus 14-deoxyandrographolide and 14-deoxy-11,12-didehydroandrographolide together (12 ± 3%), showed potent immunomodulatory activity in both in vivo and in vitro experimental models by modulating the altered immune responses during antigen interaction and ameliorating cyclophosphamide-induced immune suppression. Interestingly, over 30 days of HN-02 administration in mice, the total WBC count and the relative weight of the spleen and thymus significantly increased, supporting the argument that the substance could stimulate the humoral and cellular pathway of the immune system, both directly and indirectly [132].

A recent study evaluating the potential of *A. paniculata* as an immunostimulant for COVID-19 patients using a combination synergy analysis, based on network pharmacology, suggested that the compounds in *A. paniculata* possessed immune-protective and antiviral properties via different pathways, including the toll-like receptor pathway, the PI3/AKT pathway, and the MAP kinase pathways, which could offer benefits against SARS-CoV-2 and upper respiratory tract infections [133].

#### 3.4.2. Anti-Inflammatory Activity

In severe cases of SARS-CoV-2 infection, pulmonary inflammation, as well as respiratory failure from acute respiratory distress syndrome (ARDS), and multiorgan failure, are commonly found and have been identified as the major causes of death. An exaggerated release of pro-inflammatory cytokines, such as TNF-α, IL-6, IL-1, IL-8, and MCP-1, or “cytokine storm syndrome”, has been observed in severe COVID-19 patients. The rise of pro-inflammatory mediators (e.g., prostaglandins and leukotrienes) or a so-called “macrophage-derived eicosanoid storm” have also been detected, leading to the induction of hyperinflammation and mortality [4,134]. Since viral-induced hyperinflammation is known to affect disease progression and prognosis, the resolution of inflammation is a necessary strategy, in addition to anti-viral agents, in the management of critical COVID-19 patients who are suffering from a cytokine storm [4,135]. Moreover, a recent review showed that several herbal medicines used in acute respiratory infection treatment possessed anti-inflammatory activity and some of them showed favorable in vitro activities against SARS-CoV-2 induced inflammation via different pathways, such as by reducing pro-inflammatory cytokines and suppressing NF-kB [5,136].

*A. paniculata* extract and its bioactive components, as well as the related synthetic compounds, have been studied for their anti-inflammatory activities against endogenous or exogenous causes. Different mechanisms of action were proposed as being responsible for the anti-inflammatory activity of a major compound, andrographoide [24], such as inhibiting intercellular adhesion molecule-1 (ICAM-1) expression and endothelial-monocyte adhesion, induced by tumor necrosis factor-α (TNF) [137], down-regulating the PI3K/Akt signaling pathway, and down-streaming target nuclear factor (NF)-κB activation [138], reducing proinflammatory proteins by blocking the DNA binding of NF-κB [139], suppressing NF-κB and nitric oxide (NO) [85], and modulating macrophage and neutrophil activity [10]. Not only andrographolide but also the diterpenoids isolated from *A. paniculata*, namely, dehydroandrographolide and neoandrographolide, also exhibited anti-inflammatory activities by affecting cyclooxygenase (COX)-1 and -2 and down-regulating the expression of genes associated with inflammation response, including cytokines and cytokine receptors, chemokines, JAK/STAT signaling, TLRs family, and NF-κB [140]. Moreover, the crude extract of *A. paniculata* showed potent inhibitory activities on pro-inflammatory (NO, IL-1 beta, and IL-6) and inflammatory (PGE2 and TXB2) mediators [141]. Several studies have been conducted, suggesting that the anti-inflammatory effect of *A. paniculata* extract and pure compounds were beneficial in breast, colon, and lung cancer, rheumatoid arthritis, and angiogenesis [85]. The anti-inflammatory properties of andrographolide and the related compounds also suggested protective effects against toxicity in several organs and cells, such as cyclophosphamide (CTX)-induced intestinal toxicity [142], lipopolysaccharide-induced neurotoxicity, and liver and hepatorenal toxicity [10,143]. In these studies, the lowered levels of proinflammatory cytokine TNF-alpha and other cytokines, such as IFN gamma, IL-2, and GMCSF, were observed [142].

Interestingly, a recent in vivo study on the anti-influenza activity of andrographolide in post-infection treatment revealed that andrographolide improved the survival rate and lung pathology, as well as demonstrating decreased viral loads and the expression of inflammatory cytokines via NF-kB and the JAK-STAT signaling pathway [144]. Moreover, several studies also reported that andrographolide possessed anti-pulmonary inflammation activity [7]. An in vivo assay using a mouse allergic asthma model concluded that andrographolide could reduce allergen-induced inflammation, cellular infiltration in the airway, and airway hyper-responsiveness by inhibiting NF-κB expression in the lung and suppressing the NF-κB expressed in the nucleus of airway epithelial cells [145]. According to the study by Tan et al., andrographolide prevented lung inflammation, induced by non-typeable *Haemophilus* influenza infection (NTHi) in a cigarette smoke-exposed mouse model, by decreasing lung cellular infiltrates and the expression of cytokines and chemokines, including TNF-α, IL-1β, CXCL1/KC, 8-OHdG, matrix metalloproteinase-8 (MMP-8), and MMP-9, as well as regulating the expression of Nrf2 and its downstream genes [146]. Ko et al. examined nine Chinese traditional medicines that are typically used in inflammation and viral infection and found that *A. paniculata* extracts showed the greatest potency in inhibiting the secretion of RANTES, a chemotactic cytokine, in influenza A virus (H1N1)-infected human bronchial epithelial cells (A549) with an IC_50_ of 1.2 ± 0.4 μg/m [147]. In addition, andrographolide sulfonate, a water-soluble form of andrographolide, could improve acute lung injury (ALI) induced by lipopolysaccharide (LPS), via NF-κB and the MAPK-mediated inflammatory responses [148].

In light of the anti-inflammatory activities of *A. paniculata,* andrographolide and the related compounds that involve various types of targets and pathways, including cytokines, chemokines, adhesion molecules, nitric oxide, lipid mediators, transcription factors NF-κB, AP-1, and HIF-1, and signaling pathways such as PI_3_K/Akt, MAPK, and JAK/STAT [149,150], together with its anti-pulmonary inflammation property, *A. paniculata* could be a promising herbal medicine that should be considered as an alternative or complementary treatment to alleviate COVID-19 disease progression.

#### 3.4.3. Cold/Flu

*A. paniculata* has been widely used in many countries, especially in China, India, Southeast Asia, and Scandinavia, from generation to generation for the prevention and treatment of the common cold, flu, and related symptoms. Scientific data has revealed that *A. paniculata* and its active constituents show significant antipyretic activity. An in vivo study reported that *A. paniculata* ethanolic extract at 500 mg/kg, administered via an intragastric route, showed a significant capability in reducing fever that was comparable to that of 200 mg/kg of a standard treatment, aspirin [151], while 300 mg/kg oral doses of andrographolide presented an antipyretic activity that was comparable to the same amount of aspirin [152]. Although a previous study from Deng discovered that andrographolide, neoandrographolide, and dehydroandrographolide were active constituents in relieving fever [153], a recent study found that the synthetic compounds 3,19-isopropylidenyl-andrographolide and 3,19-dipalmitoyl-14-deoxy-11,12-didehydroandrographolide were more active than their parent compounds, andrographolide and 14-deoxy-11,12-didehydroandrographolide, in terms of antipyretic as well as analgesic and anti-inflammatory activity, with no serious toxicity at the administered doses. The increment of lipophilicity was suggested to improve the pharmacokinetic properties [154].

A number of clinical studies were carried out that confirm the efficacy of *A. paniculata,* in the form of dried leaves or commercial preparations against flu and the common cold, associated with uncomplicated acute upper respiratory tract infection in different target groups [155,156,157,158]. This effectiveness might be attributed to the immunomodulatory and anti-inflammatory activities of *A. paniculata* [10]. Systematic reviews and the meta-analyses of randomized controlled trials suggest that the use of *A. paniculata* extract alone or in combination was beneficial in early treatment or as an alternative therapy for uncomplicated acute upper respiratory tract infection. *A. paniculata* was superior to the placebo in terms of reducing the severity of uncomplicated acute upper respiratory tract infections [159,160]. The mean difference in the reduction in symptom severity scores was 2.13 points (95% CI 1.00–3.26 points, *p* = 0.0002). The difference in effect between *A. paniculata* and the placebo was 10.85 points (95% CI 10.36–11.34 points, *p* < 0.0001). A high dose of *A. paniculata* was reported to be more active than a low dose, with no serious side effects [159]. These results are consistent with those of a recent systemic review and meta-analysis involving 33 randomized controlled trials (7175 patients). The study identified that *A. paniculata,* alone or in combination with standard therapy, was superior to the placebo in terms of improving acute upper respiratory tract infection (ARTI) symptoms, including cough (*n* = 596, standardized mean difference (SMD): −0.39, 95% confidence interval CI (−0.67, −0.10)) and sore throat (*n* = 314, SMD: −1.13, 95% CI (−1.37, −0.89)). Moreover, when compared to the placebo, standard treatment, and other alternative herbal remedies, *A. paniculata* was more effective in alleviating the overall ARTI symptoms. Using *A. paniculata* could also reduce the duration of coughs and sore throats, and shortened the recovery time when compared to standard therapy [161]. According to existing data from the clinical study and systematic reviews, *A. paniculata* was considered a promising and safe natural product in the management of ARTI. However, variations in the active constituents due to the origin of the plant and the part used, as well as the quality of manufacturing and the standardization of preparation, have raised controversial issues regarding the quality of existing studies and any recommendation regimen of *A. paniculata* preparations. So far, no serious adverse events have been reported; only a few cases of mild and infrequent adverse events were observed, including unpleasant sensations in the chest [159], intensified headaches, or gastrointestinal side effects [161]. More details on the adverse effects will be discussed later in the following section.

### 3.5. Safety Data of Andrographis paniculata

#### 3.5.1. Adverse Effects

The administration of *A. paniculata* at the usual recommended dosage showed safety and good tolerability, with no adverse effects reported during the studies [157,162,163,164,165,166]. Adverse effects reported in the short-term studies of the clinical trial have been mild, infrequent, and spontaneously recovered from [155,159,167,168]. Thamlikitkul and his Thai colleagues performed a trial on 152 patients with pharyngotonsillitis symptoms, randomized into three groups to receive a low dose of *A. paniculata* (3 g/day), a high dose (6 g/day), and paracetamol as placebo control [155]. Side effects found in this study included nausea, vomiting, abdominal discomfort, dizziness, drowsiness, and malaise. Among the clinical trial of *A. paniculata* products sold in the markets, the incidence of adverse effects was reported; for example, the adverse effects of KalmCold™ (total andrographolide, 38.40% *w*/*w*) were epistaxis, diarrhea, urticaria, and vomiting [167], while for Paractin^®^ (total andrographolide, 30% *w*/*w*), they were headache, diarrhea, nausea, stomach discomfort, fatigue, cramp, and pruritus/rash [169]. Furthermore, in a phase I clinical trial study conducted on 13 HIV-positive patients and 5 healthy volunteers, the andrographolide regimen was 5 mg/kg for 3 weeks, increasing to 10 mg/kg for 3 weeks, and to 20 mg/kg for a final 3 weeks [110]. The adverse events investigated during the trial were diarrhea, dizziness, dysgeusia, eyes becoming sensitive to the light, fatigue, headache, heartburn, lymphadenopathy, nausea, pruritus/rash, tender lymph nodes, a decreased sex drive, decreased short-term memory, and anaphylactic reaction. The VigiAccess database (www.vigiaccess.org, accessed on 11 March 2020)), a platform for the general public to access information regarding pharmacovigilance and medicine safety, managed by the WHO Collaborating Center for International Drug Monitoring, Uppsala Monitoring Center (UMC) showed the reports of adverse reactions of *A. paniculata* usage in a total of 274 records from 2004–2020. The adverse events most frequently reported were: skin and subcutaneous tissue disorders, i.e., urticaria, rash, pruritus (183 reports), immune system disorders, i.e., anaphylactic reaction, hypersensitivity (37 reports), general disorder, and administration site conditions, i.e., face edema, fatigue, chest discomfort (34 reports), gastrointestinal disorders, i.e., nausea, vomiting, diarrhea, lip swelling (32 reports), and others. Focusing on the acute hypersensitivity reaction, Farah et al. suggested that the product label should contain warnings about the possibility of allergies from *A. paniculata*-based products [170].

#### 3.5.2. Toxicity

Usually, a traditional dose of *A. paniculata* powder is 9–15 mg, once a day, which contains andrographolide at an amount of 90–150 mg [155]; in Chinese traditional medicine and orthodox medicine in Thailand and India, this has long been recognized as a safe amount [8]. However, when determining the safety of patients, scientists attempted to establish the toxicity data of *A. paniculata*. Toxicity studies were designed with both animals and humans. When conducted in HIV-infected patients and healthy volunteers, no toxicity was observed in an acute toxicity study of dose-escalating andrographolide, at 5 mg/kg body weight and three times a day (TID) for 3 weeks, 10 mg/kg body weight TID for a further 3 weeks, and 20 mg/kg body weight for a final 3 weeks [110]. In the animal model, Panossian et al. reported that the oral administration of *A. paniculata* standardized extract (andrographolide, 4.6%, and 14-deoxo-andrographolide, 2.3%) in doses of 200, 600, and 2000 mg/kg given to Wistar rats did not show toxicity [171]. Moreover, in female rats treated with standardized *A. paniculata* alcoholic extract (andrographolide > 30% *w*/*w*) at 5000 mg/kg, there were no treatment-necessitating toxic effects [172]. In 2009, it was revealed that mice that were intraperitoneally administered isopropylidene andrographolide and 14-deoxy-11,12-didehydro-3,19 dipalmitoyl andrographolide at 0.5, 1, 4, 8, 50, or 100 mg/kg were able to survive [154]. Bothiraja et al. reported that although the mice were treated with andrographolide at the maximum dose (5 g/kg), no dead mice were found [173]. Oral administration of the first true-leaf ethanolic extract of *A. paniculata* contained high levels of 14-deoxy-andrographolide but low andrographolide levels at 5000 mg/kg in mice; the results showed that all treated animals survived [174]. The median lethal dose (LD_50_) of *A. paniculata* extract and its andrographolide was tested in several studies, as shown in Table 2. In order to perform a subacute toxicity test, Wistar rats consumed andrographolide at 250 mg and 500 mg/kg for 21 successive days [173]. The result demonstrated that significant alterations of behavior, biochemicals, body weight gain, food intake, hematology, histopathology, mortality, and vital organ weight were undetectable.

#### 3.5.3. Contraindication

*A. paniculata* is widely used in many countries at all stages of human life for various ailments. However, the scientific data that confirm the safety of usage of this herb among children, pregnant and lactating women, and the elderly are still ambiguous. In Ayurvedic traditional medicine, it was declared that *A. paniculata* is edible during pregnancy for a short time. To support the previous information, in 1999, pregnant rats in the first 19 days of pregnancy consumed extracts of *A. paniculata* leaves in 200, 600, and 2000 mg/kg doses (a higher dose than the usual therapeutic dose in humans at 30-, 90-, and 300-fold, respectively), undergoing an investigation of the content of the blood progesterone hormone, a necessary hormone for gestation [171]. The alteration of progesterone levels in the blood plasma of rats was not found; it was assumed that the human therapeutic dose of *A. paniculata* extract did not have an effect on the progesterone-mediated termination of pregnancy. Conversely, Satika et al. revealed that 85 female rats exposed to the water extract of *A. paniculata* (1 g/kg) for 4, 6, and 8 weeks exhibited a reduction in the female sex hormones: follicle-stimulating hormone, luteinizing hormone, estrogen, and progesterone, in a time-dependent fashion [179]. Zoha et al. studied the antifertility of *A. paniculata* in mice [180]. In a study giving 2 g per kilogram of the dried *A. paniculata*, orally fed only to female mice daily for six weeks, after mating, there were no pregnant mice. This result implies that *A. paniculata* probably inhibits ovulation in female mice. Furthermore, Noordalilati and other Malaysian colleagues (2005) found that when a 50% ethanolic extract of *A. paniculata* (andrographolide 1.33%) at 10 and 100 mg/kg of extracts, fed from day 6 to day 15 of pregnancy to Sprague Dawley rats, potentially teratogenic and toxic effects to the fetuses were related to abnormal features in the fetuses, such as micrognathia and exencephaly [181]. However, there is no information regarding the clinical trial study on pregnant women. Therefore, the usage of *A. paniculata* during pregnancy should be avoided, especially during early pregnancy [182]. In addition, information regarding its safety among breastfeeding women, children, and the elderly is insufficient; therefore, these groups should not use it, nor should those people who are allergic to plants in the Acanthaceae family [11].

#### 3.5.4. Drug Interactions

Because *A. paniculata* has been commonly used in various counties in Asia and Europe, the interaction between this herbal medicine and modern drugs, which may cause adverse drug reactions (ADRs) and toxicities, brings up numerous safety concerns. In the metabolism process of xenobiotics and drugs, there are several enzymes related to the metabolic steps in phase I, i.e., cytochrome P450 (CYP), and phase II, i.e., UDP-glucuronosyltransferase, sulfotransferase, and glutathione-*S*-transferase [183]. The concomitant administration of *A. paniculata* with drugs will probably induce or inhibit the metabolic enzymes in the intestine and liver and affect the pharmacokinetic changes. The extract of *A. paniculata,* as well as its major compound, andrographolide, underwent studies on their interaction with the CYP family, both in vitro and in vivo (Table 3). It has been reported that approximately 50% of the drug metabolism is taken up by CYP3A4 [184]. In the in vitro studies, it was found that the ethanolic and methanolic extracts, andrographolide and 14-deoxy-11,12-didehydroandrographolide, inhibited the activity of the CYP 3A4 isozyme [185,186,187]. As a consequence, the concurrent usage of *A. paniculata* with prescribed medicines that are metabolized by CYP3A4 (i.e., chemotherapeutics, antihistamine, benzodiazepine, calcium channel blocker, and statins) causes the potential risk of toxicity. Drugs with a narrow therapeutic index, such as digoxin, phenytoin, theophylline, and warfarin are drugs with a small difference between therapeutic and toxic levels; therefore, a small fluctuation in drug concentration in the blood circulation can lead to serious therapeutic failure or adverse drug reactions. The metabolism of theophylline and warfarin is involved with the CYP1A2 enzyme. It is reported that in the in vitro studies, an ethanolic extract of *A. paniculata* and andrographolide, with its derivative, exhibited the inhibition of CYP1A2 activity [185,188]. Therefore, taking these medications with the herb probably interacts with the metabolism, which is a contributor to the potential risk of toxicity. Furthermore, *A. paniculata* and its major compounds are able to inhibit the CYP2C isoform (CYP2C9, 2C11, 2C19), and CYP 2D6, as shown in Table 3. On the other hand, some studies have revealed that the alcoholic extract and andrographolide can induce activities in the CYP1A1 and CYP2B isozyme, both in vitro and in vivo ([188,189,190,191]). In phase II drug metabolism, it was found that both the ethanolic and methanolic extracts, including andrographolide and the derivatives, showed the inhibition of UDP-glucuronyltransferases or the UGT isoforms (UGT1A3, UGT1A8, UGT2B7, UGT1A1, UGT1A6, UGT1A7, and UGT1A10) [192,193,194].

Pharmacokinetic and pharmacodynamic studies of the interaction between *A. paniculata* and various therapeutic drugs were performed, both in vitro and in vivo (Table 3). The pharmacokinetics of warfarin, one of the narrow therapeutic-index drugs, was investigated in male Sprague-Dawley rats that were co-administered with warfarin (0.5 mg/kg) and andrographolide (30 mg/kg/day for 7 days) [195]. It is reported that andrographolide was able to increase the systemic level of warfarin via interference with enzymes in the metabolic process. Moreover, Chien and Chinese colleagues revealed that the phytochemical components in *A. paniculata* possibly interact with theophylline and inhibit CYP1A2, causing a delay in drug elimination [196]. In terms of the interaction between *A. paniculata* and non-steroidal anti-inflammatory drugs (NSAIDs), it was found that andrographolide demonstrates anti-arthritic activity through the mechanism of reducing pro-inflammatory mediators (COX-2, iNOS, and cytokines) [197]; therefore, the andrographolide manifested synergistic anti-arthritic activity with NSAIDs, such as naproxen [198] and etoricoxib [199]. Conversely, co-administered *A. paniculata* extract and andrographolide with nabumetone showed a reduction in anti-arthritic activity [198]. In addition, andrographolide feasibly influenced the CYP1A2 enzyme in the metabolism of NSAIDs. Only one study was performed on the pharmacokinetic and pharmacodynamic interaction of andrographolide and hypoglycemic drugs. Samala and Veeresham reported that the concurrent use of andrographolide and glyburide was capable of changing the pharmacokinetic and pharmacodynamic parameters because andrographolide contributes to the inhibition of CYP3A4, which is responsible for glyburide metabolism and improves absorption [200]. It was found that andrographolide demonstrated the inhibition of tumor growth [201], the suppression of colitis-associated colon cancer [202,203], and the induction of apoptosis in different cancer cells [204], as well as strengthening the cytotoxic effect of several chemotherapy drugs [62,205]. In 2009, Yang et al. reported that andrographolide can synergistically induce the apoptosis of 5-fluorouracil (5-FU) via the augmentation of caspase-8, p53 activity, and the significant alteration of the Bax conformation in hepatocellular carcinoma (SMMC-7721) [206]. In a subsequent study, it was reported that andrographolide exhibits an increase in apoptosis and Bax level when treated with 5-FU in human colorectal cancer [207]. In addition, an investigation was also performed on andrographolide’s effect on anticancer activity in non-small-cell lung cancer (NSCLC) (A549), co-treated with paclitaxel. The combination of andrographolide and paclitaxel showed a significant synergistic anticancer effect on A549 cells in vitro and in vivo because of the accumulation of reactive oxygen species [208]. Studies of the anticancer effect of the co-administration of andrographolide and cisplatin in the cisplatin-resistant ovarian cancer cell line A2780^cisR^ found that the synergism of anticancer activity and the percentage of apoptotic cell death in ovarian cancer cell lines was observed [209].

## 4. Discussion

Since the initial rapid outbreak and the spread of COVID-19, scientists around the world have been searching for a vaccine. Medicines utilizing both chemicals and herbal substances have been sought to decrease the number of cases and eliminate this epidemic. *A. paniculata* is one of the most popular therapeutic herbs and is recommended in combination with modern medicine in many countries, such as China and Thailand. For a limited period of time, in silico studies have been the most widely used method for studying the effects of *A. paniculata* and its constituents. The therapeutic targets were divided into two parts: virus-specific target proteins and human-specific target proteins. It was discovered that the active ingredient isolated from *A. paniculata* was more likely to act on virus-specific target proteins. In particular, 3CLpro was investigated in silico, and the results were confirmed by an in vitro study. However, the results showed that the IC_50_ of andrographolide when inhibiting 3CLpro was greater than its IC_50_ when inhibiting infectious virion production [97,101]. Therefore, it was hypothesized that andrographolide was likely to act on other virus-specific target proteins as well; however, further in vitro and in vivo studies will be needed to confirm this hypothesis. When looking at the results of a study on hACE2 where human-specific target proteins were examined, it was reported that andrographolide binds to hACE2 with some instability, while the docking binding energy does not reach the cut-off value that should be reached for a potential compound [91]. This finding also contradicts another study reporting that andrographolide stably binds to hACE2 [81]. As a result, more research on the influence of *A. paniculata* and its constituents on hACE2 is needed, particularly via in vitro and in vivo trials.

Although the scientific evidence behind using *A. paniculata* to treat patients who have suffered from COVID-19 is still unclear, the national health committees of some countries have attempted to use *A. paniculata* as a supportive therapy to cope with the pandemic situation. For example, in Xiyanping, China, a marketed injection of *A. paniculata* extract containing andrographolides has been suggested in the China National Health Commission guidelines, to treat patients who have been infected with COVID-19 in progressive stages [214]. The Xiyanping injection program showed reductions in inflammation and the improvement of virus clearance and related symptoms, such as cough, fever, and rales in the lungs [214,215]. Recently (June 2021), *A. paniculata* has been listed in the guidelines of the National List of Essential Medicines (NLEM) to manage the COVID-19 pandemic in Thailand. The oral dosage (pills and capsules) of *A. paniculata* and its extract, with andrographolide at 180 mg per day, was recommended for patients infected with COVID-19 and exhibiting mild symptoms to avoid progression to intensive symptoms [12]. In India, the Ministry of AYUSH launched a recommendation to utilize *A. paniculata* in an Ayurvedic procedure for COVID-19 treatment and to boost immunity [216]. These efforts are intended to support the policies of the national health care systems, to resolve the problematic situations arising from COVID-19 in those countries. A recent clinical trial conducted in Thailand showed that 180 mg of *A. paniculata* extract, taken daily for five consecutive days, helped to reduce the symptoms and duration of COVID-19 more successfully than andrographolide at 60 mg/day and standard treatment, with mild adverse effects (diarrhea and stomach ache) [217]. 

## 5. Conclusions

In conclusion, in situations where modern medicines and herbal medicines are urgently needed, *A. paniculata* could serve as a promising source of lead compounds for drug discovery in the post-infectious treatment of COVID-19, rather than a prophylactic. In the meantime, the use of *A. paniculata* products should be given under the supervision of physicians or pharmacists, in terms of its toxicity, adverse effects, use by specific people, and allergies, including interactions between *A. paniculata* and modern medicines. Furthermore, in light of the high demand for *A. paniculata* in the context of health benefits in this situation, the quality of *A. paniculata* products, in terms of their safety and efficacy, is inevitably a matter of concern.

## Figures and Tables

**Table 1:** Chemical constituents of *Andrographis paniculata*: *ent*-labdane diterpenoids, flavonoids, and other substances.

Chemical Substances	Parts *	References
(13*R*,14*R*) 3,13,14,19-tetrahydroxy-*ent*-labda-8(17),11-dien-16,16-olide	LV	[39]
12-*epi*-14-deoxy-12-methoxyandrographolide	AP AP AP	[21] [40] 13]
12-hydroxyandrographolide	AP	[40]
13,14,15,16-tetranor-*ent*-labda-8(17)-ene-3,12,19-triol	AP	[41]
14-deoxy-11-hydroxyandrographolide	AP	[42]
14-deoxy 11,12-didehydroandrographolide	WP LV AP LV AP AP LV AP AP AP LV AP AP	[43] [44] [41] [45] [46] [21] [13] [47] [48] [40] [49] [42] [50]
14-deoxy-11,12-didehydroandrographiside	AP AP LV AP AP	[21] [41] [13] [51] [42]
14-deoxy-11,12-dihydroandrographiside	AP	[40]
14-deoxy-11,12-dihydroandrographolide	LV, ST LV WP WP	[37] [52] [53] [34]
14-deoxy-11,14-didehydroandrographolide	AP	[54]
14-deoxy-11-hydroxyandrographiside	AP	[42]
14-deoxy-11-hydroandrographolide	LV	[37]
14-deoxy-11-hydroxyandrographolide	AP AP WP AP	[21] [13] [53] [40]
14-deoxy-11-hydroxy-*ent*-labda-8(17),12-diene-16,15-olide	LV	[37]
14-deoxy-11-oxoandrographolide	WP LV AP AP AP	[43] [45] [46] [40] [42]
14-deoxy-12-hydroxyandrographolide	AP AP LV AP	[21] [13] [52] [40]
14-deoxy-12-methoxyandrographolide	LV AP AP AP WP	[45] [21] [13] [40] [34]
14-deoxy-14,15-didehydroandrographolide	AP	[54]
14-deoxy-15-isopropylidene-11,12 didehydroandrographolide	LV WP AP	[13] [53] [47]
14-deoxy-15-methoxyandrographolide	AP	[55]
14-deoxy-17-hydroxyandrographolide	AP	[40]
14-deoxy-8,17-epoxy-andrographolide	AP	[42]
14-deoxyandrographiside	LV AP AP LV	[37] [46] [42] [39]
14-deoxyandrographolide	CL WP AP LV AP AP LV LV LV WP AP AP AP WP LV	[56] [43] [41] [45] [46] [21] [13] [44] [52] [53] [48] [40] [42] [34] [39]
14-deoxyandrographolide-19-β-glucoside	N/A	[57]
14-deoxyandrographoside	N/A	[57]
14-*epi*-andrographolide	AP LV	[21] [13]
15-methoxy-3,19-dihydroxy-8(17)11,13-*ent*-labda-trien-16,15-olide	AP	[58]
15-spiro diterpenoids dimer bisandrographolide G	AP	[59]
19-[(β-d-glucopyranosyl)oxy]-19-oxo-*ent*-labda-8(17),13-dien-16,15-olide	AP	[41]
19-hydroxy-3-oxo-*ent*-labda-8(17),11,13-trien-16,15-olide	AP	[41]
19-hydroxy-*ent*-labda-8(17),13-dien-15,16-olide	AP	[41]
19-nor andrographolide A-C	AP	[60]
19-*O*-β-d-glucopyranosyl-*ent*-labda-8(17),13-dien-15,16,19-triol	AP	[51]
19-O-[β-d-apiofuranosyl-β-d-glucopyranosyl]-3,14-dideoxyandrographolide	AP	[50]
19-*O*-[β-d-apiofuranosyl(1→2)-β-d-glucopyranoyl]-3,14-dideoxyandrographolide	AP	[40]
19-*O*-β-d-glucopyranosyl-*ent*-labda-8(17),13-dien-15,16,19-triol	AP AP	[42] [50]
19-*O*-[β-d-apiofuranosyl(1→2)-β-d-glucopyranoyl]-3,14-dideoxyandrographolide	AP	[40]
21-nor-3,19-isopropylidine-14-deoxy-*ent*-labda-8(17),13-diene-16,15-olide	LV	[52]
3-*O*-β-d-glucopyranosylandrographolide	AP	[40]
3,13,14,19-tetrahydroxy-*ent*-labda-8(17),11-dien-16,15-olide	LV	[39]
3,14-deoxy-17β-hydroxy andrographolide	AP	[42]
3,14-dideoxyandrographolide	AP AP	[41] [46]
3,15,19-trihydroxy-*ent*-labda-8(17),13-dien-16-oic acid	AP	[41]
3,18,19-trihydroxy-*ent*-labda-8(17),13-dien-16,15-olide	AP LV	[41] [37]
3,19-dihydroxy-14,15,16-trinor-*ent*-labda-8(17),11-dien-13-oic acid	AP LV	[41] [37]
3,19-dihydroxy-15-methoxy-*ent*-labda-8(17),11,13-trien-16,15-olide	AP	[41]
3,19-dihydroxy-*ent*-labda-8(17),12-dien-16,15-olide	AP AP	[42] [41]
3,19-isopropylidene-14-deoxy-*ent*-labda-8(17),13-diene-16,15-olide	LV	[39]
3-deoxy-andrographoside	AP	[55]
3-*O*-β-d-glcopyranosyl 14,19-dideoxyandrographolide	AP	[40]
3-*O*-β-d-glucopyranosylandrographolide	AP	[40]
3-*O*-β-d-glucosyl-14-deoxy-11,12-didehydroandrographiside	AP	[61]
3-*O*-β-d-glucosyl-14-deoxyandrographiside	AP	[62]
3-oxo-14-deoxy-11,12-didehydroandrographolide	AP	[58]
3-oxo-14-deoxyandrographolide	AP	[58]
3-*O*-β-d-glucopyranosyl-14,19-dideoxyandrographolide	AP	[40]
3-*O*-β-d-glucopyranosylandrographolide	AP	[42]
3-*O*-β-d-glucosyl-14-deoxy-11,12-didehydroandrographiside	AP	[62]
3-*O*-β-d-glucosyl-14-deoxyandrographiside	AP	[62]
5-hydroxy-7,20,60-trimethoxyflavone	RT	[13]
6ʹ-acetylneoandrographolide	AP AP	[21] [42]
8,17-epoxy-14-deoxyandrographolide	AP	[40]
8-methylandrographolide	LV	[39]
8α-methoxyl-14-deoxy-17β-hydroxyandrographolide	AP	[63]
Andrograpanin	CL LV LV LV LV AP AP RT	[56] [45] [64] [65] [44] [48] [40] [66]
Andrographane	LV	[13]
Andrographatoside	AP	[40]
Andrographic acid	N/A	[67]
Andrographidine A	RT N/A RT AP	[68] [67] [66] [35]
Andrographidine B	RT RT	[68] [66]
Andrographidine C	RT RT AP	[68] [66] [35]
Andrographidine D	RT	[68]
Andrographidine E	RT	[68]
Andrographidine F	RT	[68]
Andrographidoids A–E	RT	[69]
Andrographiside	AP AP AP AP AP AP AP	[41] [46] [21] [51] [40] [42] [50]
Andrographolactone	LV, ST AP	[37] [70]
Andrographolide	N/A LV LV CL N/A AP AP AP AP AP WP LV WP AP AP AP LV LV AP WP LV RT	[18] [45] [52] [56] [71] [41] [54] [72] [46] [21] [13] [44] [53] [47] [48] [40] [49] [73] [42] [34] [39] [50]
Andrographone	LV	[13]
Andrographoside	N/A WP LV	[57] [53] [39]
Andropaniculoside A	WP	[34]
Andropaniculosin A	WP	[34]
Andropanioside A–B	AP	[51]
Andropanolide	LV LV AP	[45] [44] [51]
Andropanoside	AP	[40]
Bisandrographolide A	AP	[21]
Bisandrographolide B	AP AP	[21] [40]
Bisandrographolide C	AP AP	[21] [40]
Bisandrographolide D	AP	[21]
Bisandrographolide ether	AP	[48]
Dehydroandrographolide	AP	[50]
Deoxyandrographiside	AP AP WP AP	[21] [41] [34] [50]
Deoxyandrographolide	LV	[73]
Deoxyandrographolide-19β-d-glucoside	LV	[73]
Dihydroxyl dimethyl 19-[(β-d-glucopyranosyl)oxy]-19-oxo-*ent*-labda-8(17),13-dien-16,15-olide	LV, ST	[37]
*ent*-labda-8(17),13-dien-15,16,19-triol	AP	[41]
Isoandrographiside	AP	[42]
Isoandrographolide	AP AP LV AP AP AP WP	[54] [21] [44] [47] [40] [42] [34]
Methyl methoxy 14-deoxyandrographiside	LV, ST	[37]
Methyl methoxy andrographolide	LV, ST	[37]
Methyl methoxy neoandrographolide	LV, ST	[37]
Neoandrographolide	CL AP LV LV AP AP AP AP AP AP LV LV AP AP LV AP WP LV RT	[56] [74] [37] [45] [54] [72] [46] [21] [13] [51] [44] [52] [47] [40] [49] [42] [34] [39] [50]
Panicolin	RT	[13]
Paniculide A	CL	[75]
Paniculide B	CL	[75]
Paniculide C	CL	[75]
Propyl neoandrographolide dimer	LV	[37]
Tetraacetate neoandrographolide	LV	[49]
Wightiolide	RT AP	[66] [42]
**Flavonoids**		
onysilin	WP	[34]
5-hydroxy-7,8-dimethoxyflavanone	AP	[44]
(2*S*)-5,2ʹ-dihydroxy-7,8-dimethoxyflavanone	AP	[54]
(2*S*)-7,8-dimethoxy-5β-d-glucopyranosyloxyflavanone	AP	[54]
2ʹ,5-dihydroxy-7,8-dimethoxyflavone-2ʹ-*O*-β-d-glucopyranoside	AP	[54]
2ʹ-hydroxy-2,4ʹ6ʹ-trimethoxychalone	ST	[37]
2ʹ-hydroxy-8,7,8-trimethoxyflavone	RT	[66]
5,2ʹ,6ʹ-trihydroxy-7-methoxyflavone 2ʹ-*O*-β-d-glucopyranoside	RT	[66]
5,2ʹ-dihydroxy-7,8-dimethoxyflavone 2ʹ-*O*-β-d-glucopyranoside	RT	[66]
5,2ʹ-dihydroxy-7,8-dimethoxyflavone	RT CL	[66] [76]
5,4ʹ-dihydroxy-7,8,2ʹ,3ʹ-tetramethoxyflavone	RT AP	[66] [35]
5,4ʹ-dihydroxy-7-methoxy-8-*O*-β-d-glucopyranosyloxyflavone	AP	[77]
5,4ʹ-dihydroxy-7-methoxyflavone-8-yl-β-d-glucopyranoside	AP	[78]
5,4ʹ-dihydroxy-7-methoxyflavone-6-yl-β-d-glucopyranoside	AP	[78]
5,4ʹ-dihydroxy-7-*O*-β-d-glucopyranosyloxyflavone	AP	[77]
5,4ʹ-dihydroxy-7-*O*-β-d-pyranoglycuronatebutylester	AP	[77]
5,5ʹ-dihydroxy-7,8,2ʹ-trimethoxyflavone	RT	[66]
5,6,4ʹ-trihydroxy-7-methoxyflavone-6-*O*-β-d-glucoside	AP	[54]
5,7,2ʹ,3ʹ-tetramethoxyflavanone	WP	[53]
5,7,2ʹ,3ʹ-tetramethoxyflavone	RT	[47]
5,7,8,2ʹ-tetramethoxyflavone	WP RT	[53] [66]
5,7,8-trimethoxydihydroflavone	AP	[77]
5-hydroxy 7,8,2ʹ,3ʹ-tetramethoxyflavone	RT WP RT	[13] [53] [66]
5-hydroxy-2ʹ,7,8-trimethoxyflavone	AP	[54]
5-hydroxy-3,7,8,2ʹ-tetramethoxyflavone	RT	[79]
5-hydroxy-7,2ʹ,3ʹ-trimethoxyflavone	WP	[53]
5-hydroxy-7,2ʹ,6ʹ-trimethoxyflavone	WP RT	[53] [78]
5-hydroxy-7,8,2ʹ,3ʹ,4ʹ-pentamethoxyflavone	RT	[66]
5-hydroxy-7,8,2ʹ,3ʹ-tetramethoxyflavone	RT	[66]
5-hydroxy-7,8,2ʹ,5ʹ-tetramethoxy (2*R*)-flavone-5-*O*-β-d-glucopyranoside	N/A	[67]
5-hydroxy-7,8,2ʹ,5ʹ-tetramethoxyflavone	AP AP RT, ST WP RT WP	[35] [54] [80] [53] [66] [34]
5-hydroxy-7,8,2ʹ,6ʹ-tetramethoxyflavone	RT	[66]
5-hydroxy-7,8,2ʹ,3ʹ-tetramethoxyflavone	AP	[35]
5-hydroxy-7,8,2ʹ-trimethoxyflavone	CL RT, ST RT	[76] [80] [66]
5-hydroxy-7,8-dimethoxy (2*R*)-flavanone-5-*O*-β-d-glucopyranoside	N/A	[67]
5-hydroxy-7,8-dimethoxyflavanone	AP RT RT	[54] [79] [66]
5-hydroxy-7,8-dimethoxyflavone	AP RT CL RT, ST AP RT	[54] [81] [82] [80] [44] [66]
7,8,2ʹ,5ʹ-tetramethoxy-5-*O*-β-d-glucopyranosyloxyflavone	AP	[77]
7,8-dimethoxy-2ʹ-hydroxy-5-*O*-β-d-glucopyranosyloxyflavone	AP	[77]
7,8-dimethoxy-5 β-d-glucopyranosyloxyflavone	AP	[54]
7-*O*-methylwogonin 5-*O*-glucoside	WP RT	[53] [78]
7-*O*-methyldihydrowogonin	LV, ST AP WP RT	[37] [77] [53] [78]
7-*O*-methylwogonin	LV, ST WP AP WP RT	[37] [34] [77] [53] [78]
Andrographidine A	RT	[68]
Andropaniculoside A	WP	[34]
Andropaniculosin A	WP	[34]
Apigenin	WP AP	[34] [77]
Apigenin-7-*O*-β-d-methylglucuronide	WP	[34]
Apigenin-7-*O*-glucoronide	LV	[37]
Cosmosiin	WP	[34]
Dihydroneobaicalein	RT	[66]
Dihydroskullcapflavone I	ST WP	[37] [53]
Diosmetin-7-glycoside	LV	[37]
Isoswertisin	WP	[34]
Luteolin	AP	[35]
Luteolin-7-*O*-glucoronide	LV	[37]
Quercetin	WP	[34]
Scutellarin-6-*O*-β-d-glucoside-7-methyl ether	WP	[34]
Skullcapflavone I 2ʹ-*O*-glucoside	WP RT	[53] [78]
Skullcapflavone I 2ʹ-methyl ether	WP RT	[53] [78]
Skullcapflavone I	WP	[34]
Skullcapflavone-2ʹ-methoxyether	AP	[77]
**Other Chemical Constituents**		
1,2-dihydroxy-6,8-dimethoxyxanthone	RT	[83]
1,8-dihydroxy-3,7-dimethoxyxanthone	RT	[83]
3,4-dicaffeoylquinic acid	WP	[34]
3,7,8-trimethoxy-1-hydroxyxanthone	RT	[83]
4,8-dihydroxy-2,7-dimethoxyxanthone	RT	[83]
4-hydroxy-2-methoxycinnamaldehyde	RT	[66]
α-sitosterol	RT	[13]
β-sitosterol	WP RT	[53] [66]
β-daucosterol	RT	[66]
Caffeoylquinic acid	ST	[37]
Caffeic acid	WP	[53]
Caffeic glycoside	LV	[37]
Chloragenic glycoside	LV WP	[37] [53]
Cinnamic acid	LV WP	[52] [53]
Coumaroylquinic acid	LV	[37]
Dihydroxyl glucosyl cyclohexane	LV, ST	[37]
Ferulic acid	WP LV	[53] [52]
Feruloylquinic acid	LV	[37]
Methyl-3,4-dicaffeoylquinate	WP	[34]
Oleanolic acid	RT	[66]
Quinic acid	LV, ST	[37]
Stigmasterol	LV	[49]
Trans-cinnamic acid	RT	[66]

* AP: aerial parts, CL: callus, LV: leaves, RT: roots, ST: stems, WP: whole plants.

**Table 2:** The median lethal dose (LD_50_) data of *Andrographis paniculata* extract and its constituents.

Substance	Route/Model *	LD_50_ Value (g/kg Bodyweight)	Reference
14-deoxy-11,12-didehydroandrographolide	oral/mice	>20	[175]
*A. paniculata* alcoholic extract	N/A	1.8	[176]
*A. paniculata* ethanolic extract (first true leaf)	oral/mice	>5	[174]
*A. paniculata* extract	N/A	>17	[177]
Andrographolide	oral/mice	>5	[173]
Andrographolide	ip/mice	11.46	[178]
Andrographolide	oral/mice	>40	[175]
Deoxyandrographolide	oral/mice	>20	[175]
Neoandrographolide	oral/mice	>20	[175]
Total diterpene lactone	oral/mice	13.4	[175]

* ip: intraperitonial injection, N/A: not available.

**Table 3:** The interaction of *Andrographis paniculata* with cytochrome P450 enzymes and the proteins associated with metabolism.

Phase I Drug Metabolism				
CYP P450 Isoform	Models	Components	Effects	Reference
CYP1A1	in vitro			
	Mouse hepatocytes	Andrographolide	significantly induce the expression of CYP1A1 mRNA	[188]
	Mouse hepatocytes	Andrographolide	induce CYP1A1 mRNA expression	[189]
	Mouse hepatocytes	Andrographolide and 11,12-didehydroandrographolide	induce CYP1A1 expression	[190]
	in vivo			
	Mouse	Aqueous and ethanolic extract of *A. paniculata*	induce CYP1A1 mRNA expression	[191]
CYP1B1	in vitro			
	Mouse hepatocytes	Andrographolide	did not affect to the expression of CYP1B1 mRNA	[188]
CYP1A2	in vitro			
	Mouse hepatocytes	Andrographolide	significantly induce the expression of CYP1A2 mRNA	[188]
	Wistar rat and human liver microsomes	Ethanolic extract of *A. paniculata* contained 1.60% andrographolide	inhibit CYP1A2 activity in rat and human liver microsomes (*Ki* value = 8.85 and 24.46 µM, respectively)	[210]
	HepG2 hepatoma cells	Andrographolide and 14-Deoxy-11,12-Didehydroandrographolide	inhibit the mRNA and protein expression of CYP1A2	[185]
CYP2B isoform	in vivo			
	Mouse	Aqueous and ethanolic extract of *A. paniculata*	induce CYP2B mRNA expression	[191]
CYP2C isoform	in vitro			
	Wistar rat and human liver microsomes	Ethanolic extract of *A. paniculata* containing 1.60% andrographolide	inhibits CYP2C both in rat and human liver microsomes with (*Ki* values 8.21 and 7.51 µM, respectively)	[210]
	Wistar rat and human hepatocytes	Ethanolic extract of *A. paniculata* containing andrographolide 50 µM	inhibit CYP2C mRNA expression and activity	[211]
	Wistar rat and human hepatocytes	Andrographolide 50 µM	inhibit CYP2C mRNA expression and activity	[211]
CYP2C9	in vitro			
	Human hepatic cytochrome P450 activities	Ethanolic extract of *A. paniculata*	inhibit CYP2C9 (*Ki* value = 17.2 ± 2.7 µg/mL)	[186]
	Human hepatic cytochrome P450 activities	Methanolic extract of *A. paniculata*	inhibit CYP2C9 (*Ki* value = 68.5 ± 12.8 µg/mL)	[186]
CYP2C11	in vivo			
	Wistar rat	Ethanolic extract of *A. paniculata* containing andrographolide 5 and 25 mg/kg/day for 28 days	diminish CYP2C11 activity	[211]
CYP2C19	in vitro			
	pCWori+ plasmid cloned with cDNA of CYP2C19	Ethanolic extract of *A. paniculata*	inhibit CYP2C19 (*Ki* value = 67.1 µg/mL)	[186]
CYP2D6	in vitro			
	HepG2 hepatoma cells	Andrographolide and 14-Deoxy-11,12-Didehydroandrographolide	inhibit the mRNA and protein expression of CYP2D6	[185]
	Human hepatic cytochrome P450 activities	Ethanolic extract of *A. paniculata*	inhibit CYP2D6 (*Ki* value = 96.5 ± 7.0 µg/mL)	[186]
	Human hepatic cytochrome P450 activities	Methanolic extract of *A. paniculata*	inhibit CYP2D6 (*Ki* value = 59.0 ± 10.6 µg/mL)	[186]
	Luminescent assay	Ethanolic extract of *A. paniculata*	inhibit CYP2D6 with IC_50_ value = 44.2 ± 4.5 µg/mL	[212]
	CYP-CO complex assay and fluorogenic assay	Methanolic extract of *A. paniculata*	inhibit CYP2D6 (IC_50_ value = 88.80 ± 3.32 µg/mL)	[213]
CYP2E1	in vitro			
	Human liver microsomes	Andrographolide	did not affect to human liver microsomes	[210]
CYP3A isoform	in vitro			
	human hepatocytes	Ethanolic extract of *A. paniculata* containing andrographolide 50 µM	decrease CYP3A mRNA expression and activity	[211]
	human hepatocytes	andrographolide 50 µM	decrease CYP3A mRNA expression and activity	[211]
CYP3A4	in vitro			
	Human liver microsomes	Ethanolic extract of *A. paniculata* contained 1.60% andrographolide	competitively inhibits on CYP3A4 (*Ki* value = 25.43 µM)	[210]
	HepG2 hepatoma cells	Andrographolide and 14-Deoxy-11,12-Didehydroandrographolide	inhibit the mRNA and protein expression of CYP3A4	[185]
	Human hepatic cytochrome P450 activities	Ethanolic extract of *A. paniculata*	inhibit CYP3A4 (*Ki* value = 9.4 ± 3.3 µg/mL)	[186]
	Human hepatic cytochrome P450 activities	Methanolic extract of *A. paniculata*	inhibit CYP3A4 (*Ki* value = 17.8 ± 2.1 µg/mL)	[186]
	Caco-2 cells	Andrographolide (1, 10, 100 µM)	down-regulates the mRNA and protein levels, and inhibits metabolic activities of nifedipine oxidation and testosterone 6β-hydroxylation	[187]
	CYP-CO complex assay and fluorogenic assay	Methanolic extract of *A. paniculata*	inhibit CYP3A4 (IC_50_ value 63.06 ± 1.35 µg/mL)	[213]
Phase II Drug Metabolism *				
**Protein**	**Models**	**Components**	**Effects**	**References**
UGT	in vitro			
	4MU glucuronidation assays	Ethanolic extract of *A. paniculata*	inhibit UGT isoforms i.e., UGT1A3, UGT1A8, UGT2B7, UGT1A1, UGT1A6, UGT1A7, and UGT1A10 (IC_50_ = 1.70, 2.57, 2.82, 5.00, 5.66, 9.55, 15.66 µg/mL, respectively)	[192]
	recombinant UGT isoforms—catalyzed 4-MU glucuronidation reaction	Andrographolide, neoandrographolide, dehydroandrographolide, deoxyandrographolide, 3-oxo-dehydroandrographolide, 9β-hydroxy dehydroandrographolide, 3-oxo-deoxyandrographolide, 9β-hydroxy deoxyandrographolide, 3-oxo-9β-hydroxy deoxyandrographolide, 3,17,19-trihydroxy-7,11,13-ent-labatrien-15,16-olide	selectively inhibit UGT2B7	[193]
	human liver microsomes	andrographolide	strongly inhibit morphine 3- and 6-glucuronidation, the substances of UGT2B7 enzyme (IC_50_ = 21.6 µM)	[194]

* UGTs: UDP-glucuronyltransferases; 4MU glucuronidation assay: 4-methylumbelliferone glucuronidation assays.

## Data Availability

Not applicable.
